# Clinical course and predictive factors of incidentally detected adult intussusceptions on CT: a retrospective cohort study

**DOI:** 10.1007/s00261-026-05531-z

**Published:** 2026-04-25

**Authors:** Abdullah Enes Ataş, Zuhal Ak, Furkan İmrek, Ülkü Kerimoğlu

**Affiliations:** Department of Radiology, Necmettin Erbakan University, Konya, Turkey

**Keywords:** Adult intussusception, Computed tomography, Transient, Conservative management, Incidental finding

## Abstract

**Background:**

Adult intussusception is traditionally considered a surgical pathology often associated with a malignant lead point. However, the widespread use of computed tomography (CT) has led to more frequent incidental detections. We aimed to evaluate the clinical and radiological characteristics of incidentally detected adult intussusceptions and identify predictors of lesion persistence.

**Methods:**

In this retrospective study, we reviewed 150 adult patients with incidentally detected intussusception on abdominal CT between 2020 and 2025. Demographic data, clinical history, and radiological parameters (location, segment length, lead point, and ileus) were recorded. In the subgroup of patients with follow-up imaging (*n* = 59), factors predicting the persistence of the intussusception were statistically analyzed.

**Results:**

The mean age was 43.4 ± 18.0 years. The majority of intussusceptions were localized in the jejunum (77.3%). A pathological lead point was identified in only 11.3% of patients, and 4.7% presented with ileus. On follow-up imaging, 74.6% of the intussusceptions resolved spontaneously, while 10.2% required surgical intervention. There was no statistically significant association between the persistence of the lesion and the length of the intussuscepted segment (*p* = 0.184), history of malignancy (*p* = 0.236), or the presence of a lead point (*p* = 0.595).

**Conclusion:**

Most incidentally detected adult intussusceptions are benign, transient, jejunal predominant processes. Traditional predictive factors, such as segment length or malignancy history, are insufficient to foresee lesion persistence. A conservative approach prioritizing clinical observation should be considered in asymptomatic patients lacking a distinct lead point or ileus.

## Introduction

Intussusception is defined as the telescopic insertion of two consecutive segments of the gastrointestinal tract into each other’s lumen [1] and is a pathology that is rare in the adult age group but, when detected, poses a serious clinical management dilemma for clinicians and radiologists [2]. Although it is common in the pediatric population and is usually idiopathic, it accounts for only 1% of all intestinal obstructions and only 5% of all intussusception cases in adults [3–5]. Traditional surgical literature suggests that 70–90% of adult intussusceptions have an identifiable pathological lead point, a significant proportion of which are malignant in origin, and therefore recommends surgical resection as the standard treatment for all identified cases [6, 7].

However, developments in radiological imaging technologies over the past twenty years, particularly in the field of Computed Tomography (CT), have significantly changed the epidemiological profile of this disease [8]. As the use of modern imaging devices with high spatial and temporal resolution increases for non-specific abdominal pain or entirely different indications (trauma, oncological screening, follow-up etc.), radiologists are encountering incidentally detected asymptomatic or mildly symptomatic intussusceptions much more frequently [9, 10]. This situation has led to the questioning of information that prioritizes surgical treatment in adult intussusception [2]. Current studies indicate that the majority of small bowel intussusceptions (enteroenteric) are transient and self-limiting physiological events resulting from abnormal peristalsis, without an underlying tumor [11, 12].

The fundamental challenge in clinical practice is distinguishing which of these lesions detected on CT require urgent surgical intervention for malignancies (adenocarcinoma, lymphoma, metastasis) or complicated benign lesions (lipoma, GIST, Meckel’s diverticulum), and which are physiological processes that will regress with conservative monitoring [13, 14]. An incorrect assessment can lead to delays in diagnosing a malignant lesion on one hand and to catastrophic events such as intestinal necrosis/perforation on the other; it can also increase the patient’s morbidity risk with an unnecessary laparotomy [15, 16].

Although childhood intussusceptions have been extensively studied in the literature, our knowledge and experience regarding adult intussusceptions is largely limited to case reports and reviews [17, 18]. Articles investigating the radiological findings, clinical follow-up, and outcomes of adult intussusceptions are fewer compared to those in the pediatric group.

This study aimed to investigate the predictive value of radiological findings on clinical outcomes based on criteria such as whether the intussusception was transient or not, the length of the intussuscepted segment, its location, and the presence of ileus in adult intussusception cases incidentally detected by CT.

## Materials and methods

### Study design and population

This retrospective study was approved by the Necmettin Erbakan University Ethics Committee (Decision No: 2025/5755, Date: 09.05.2025). The requirement for informed consent was waived due to the retrospective nature of the study. The study was conducted in accordance with the ethical standards laid down in the 1964 Declaration of Helsinki and its later amendments.

We reviewed the radiology information system (RIS) and hospital database for patients who underwent contrast-enhanced abdominal CT examinations between 1.1.2020 and 1.3.2025. The keyword “intussusception” was used to search radiological reports.

Inclusion criteria were: (1) adults aged ≥ 18 years; (2) presence of a definitive intussusception diagnosis on CT; and (3) incidental detection of intussusception (patients scanned for indications other than suspected intussusception, or where intussusception was an unexpected finding).

Exclusion criteria included: (1) patients with a known history of intussusception prior to the index CT; (2) poor image quality preventing adequate evaluation (e.g., severe motion artifacts); and (3) patients with incomplete clinical records. A total of 150 patients meeting these criteria were included in the final analysis.

### CT acquisition protocol

All CT examinations were performed using a third-generation dual-source 256-slice CT scanner (Somatom Drive, Siemens Healthineers, Erlangen, Germany). The acquisition parameters were typically set as follows: tube voltage, 100–120 kVp (using automated tube voltage selection, CARE kV); tube current, with automated current modulation (CARE Dose 4D); gantry rotation time, 0.28–0.5 s; and pitch, 0.6–0.9. Images were acquired in the portal venous phase (approximately 60–70 s after contrast injection). A non-ionic iodinated contrast medium (iohexol) was administered intravenously via an automatic power injector at a rate of 3.0–4.0 mL/s, adjusted according to the patient’s body weight (1.5–2.0 mL/kg). Reconstruction was performed with a slice thickness of 1.5 mm and an increment of 1.0 mm using a soft-tissue convolution kernel. Multiplanar reformations (MPR) in sagittal and coronal planes were automatically generated for all patients to facilitate accurate anatomical assessment.

### Image analysis

Two radiologists with 23 and 10 years of experience in abdominal imaging, blinded to the clinical outcomes, retrospectively reviewed the CT images on a picture archiving and communication system (PACS) workstation (Syngo.via, Siemens, Erlangen, Germany). In cases of discrepancy, a consensus was reached through discussion. Intussusception was defined radiologically by the presence of the pathognomonic “target” or “sausage-shaped” soft tissue mass consisting of two concentric rings of bowel loop.

For each case, the following parameters were recorded:

Location: Classified as enteroenteric (jejunal, ileal), ileocolic, or colocolic.

**Fig. 1:**
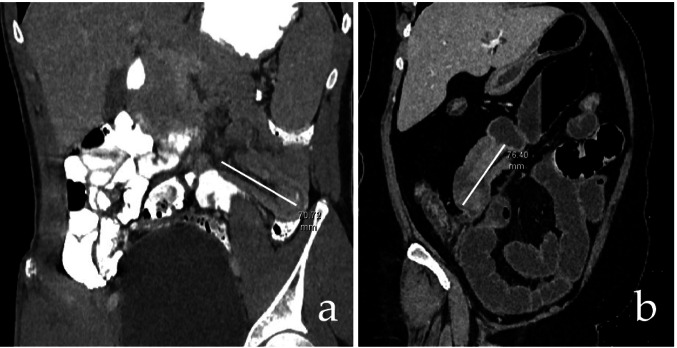
Demonstration of the measurement technique used to determine the length of the intussuscepted segment. Oblique multiplanar reconstruction (MPR) images (a, b) in different planes clearly illustrate how the maximum longitudinal dimension of the intussusceptum was calculated (white lines indicate the measured segments)

Length: The length of the intussuscepted segment was measured on multiplanar reconstructions (MPR) to ensure accuracy, measuring the maximum longitudinal dimension of the intussusceptum (Fig. 1).

Lead Point: The presence of a pathological lead point (e.g., mass, polyp, lipoma) was recorded.

Complications: Signs of intestinal obstruction (ileus), such as proximal bowel loop dilation (> 3 cm for small bowel, > 6 cm for colon) and air-fluid levels, were noted.

### Follow-up and clinical data

Demographic data (age, gender) and clinical history (history of malignancy, prior abdominal surgery, systemic comorbidities, such as diabetes mellitus, hypertension, celiac disease, inflammatory bowel disease, rheumatological disease etc.) were retrieved from electronic medical records. Follow-up data were analyzed to determine the clinical course (spontaneous resolution, persistence, or surgical intervention). “Persistence” was defined as the presence of the intussusception on subsequent imaging studies, while “resolution” was defined as the absence of the finding on follow-up CT.

### Statistical analysis

Statistical analysis was performed using IBM SPSS Statistics for Windows, Version 26.0 (Armonk, NY, IBM Corp). Visual (histograms, probability plots) and analytical methods (Kolmogorov-Smirnov/Shapiro-Wilk tests) were used to assess the normality of the data distribution.

Continuous variables were presented as mean ± standard deviation (SD) for normally distributed data and as median with interquartile range (IQR, 25th–75th percentiles) for non-normally distributed data. Categorical variables were expressed as frequencies and percentages (%).

Comparison between groups (Persistent vs. Resolved intussusception) was performed using the Mann-Whitney U test for continuous variables (age, length of segment, follow-up time) due to the non-normal distribution of the data. The Chi-square test or Fisher’s Exact test was used for categorical variables (gender, malignancy, lead point, etc.), as appropriate. A p-value of < 0.05 was considered statistically significant.

## Results

### Demographic and clinical characteristics

The study included a total of 150 adult patients. The mean age of the participants was 43.37 ± 17.97 years (median: 44.0, IQR: 28.75–58.00). There was a male predominance, with 89 (59.3%) male and 61 (40.7%) female patients. Regarding clinical history, 60 patients (40.0%) had a history of malignancy, 52 (34.7%) had a history of prior abdominal surgery, and 48 (32.0%) had systemic comorbidities (Table 1).

**Table 1:** Demographic and clinical characteristics of the patients

		n	%
Gender	Male	89	59.3
	Female	61	40.7
History of Malignancy	Yes	60	40.0
	No	90	60.0
Systemic Disease	Yes	48	32.0
	No	102	68.0
History of Surgery	Yes	52	34.7
	No	98	65.3
Location of Intussusception	Cecum-Ascending Colon	2	1.3
	Ascending Colon	3	2.0
	Hepatic Flexure and Ileal	1	0.7
	Ileal	17	11.3
	Ileocecal and Appendix	1	0.7
	Ileum-Cecum	1	0.7
	Jejunal	116	77.3
	Jejunal and Ileal	1	0.7
	Colon and Hepatic Flexure	1	0.7
	Rectosigmoid	4	2.7
	Splenic Flexure	1	0.7
	Transverse Colon	2	1.3
Ileus (Intestinal Obstruction)	Present	7	4.6
	Absent	143	95.4
Lead Point (Lesion)	Present	17	11.3
	Absent	133	88.7
Intussusception on Follow-up (*n* = 59)	Resolved (Absent)	44	74.6
	Operated	6	10.2
	Persistent (Present)	7	11.9
	Different Segment	2	3.4

The most common presenting symptoms were abdominal pain (49.3%) and incidental detection during surveillance or check-up (30.0%). Only 7 patients (4.7%) presented with clinical signs of ileus (Table 2).

**Table 2:** Distribution of presenting symptoms and indications for imaging

Presenting symptom/indication	*n*	%
Abdominal Pain	74	49.3
Follow-up/Surveillance	45	30.0
Nausea - Vomiting	6	4.0
Diarrhea	5	3.3
Hematochezia	5	3.3
Clinical deterioration	2	1.3
Flank Pain	2	1.3
Inflammatory Bowel Disease (IBD)	3	2.0
Unintentional Weight Loss	1	0.7
Anemia	1	0.7
Staging (Oncological)	1	0.7
Fever	1	0.7
Trauma	2	1.3
Altered Mental Status	1	0.7
Syncope	1	0.7
Cardiac Arrest	1	0.7

### Radiological findings

**Fig. 2:**
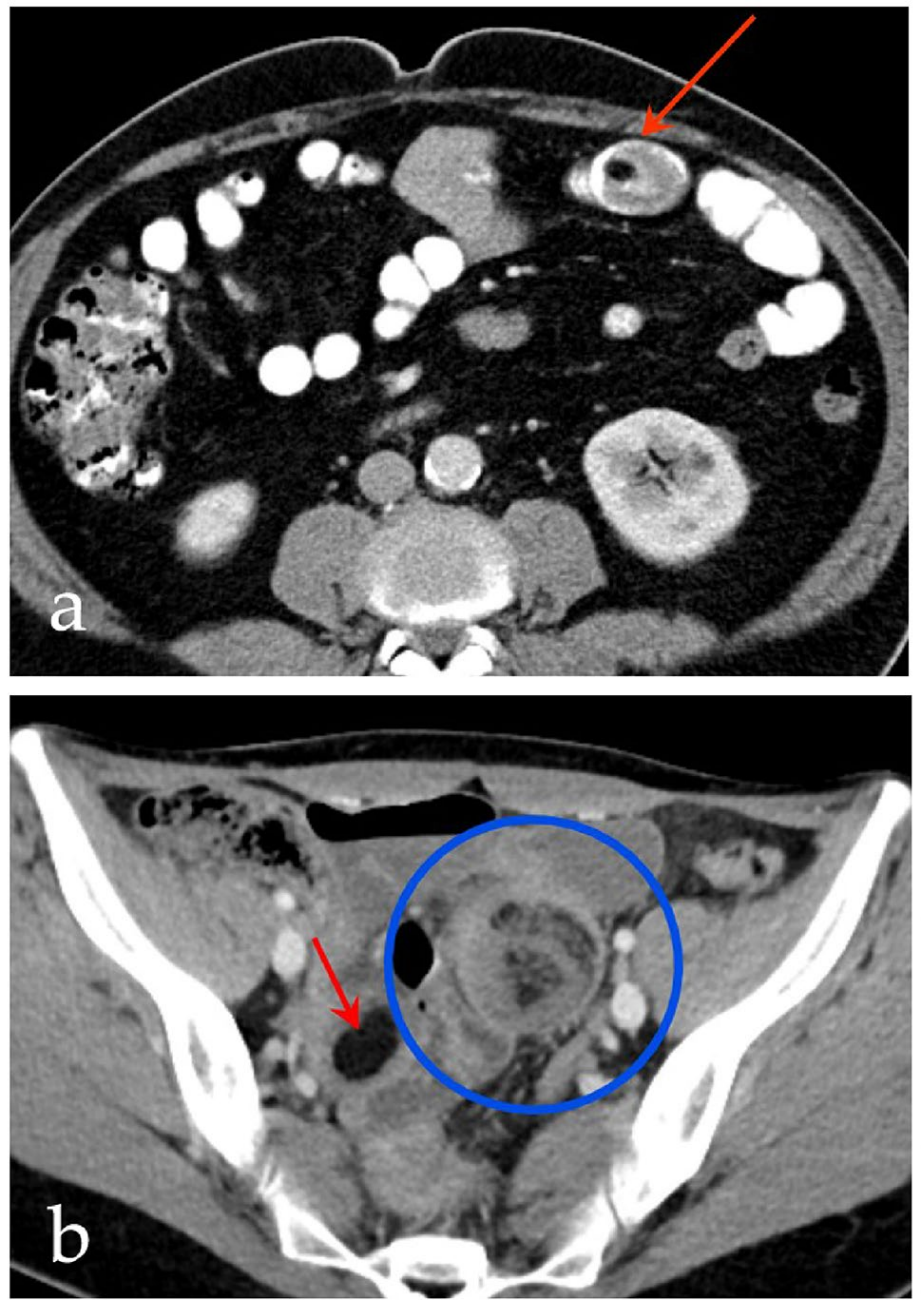
Axial contrast-enhanced CT images demonstrating adult intussusceptions caused by a benign pathological lead point. The red arrows in both panels (a, b) point to well-defined, fat-attenuating masses consistent with enteric lipomas acting as the lead points. The blue circle in panel (b) highlights the edematous, multi-layered bowel loops characteristic of the intussusception

**Fig. 3:**
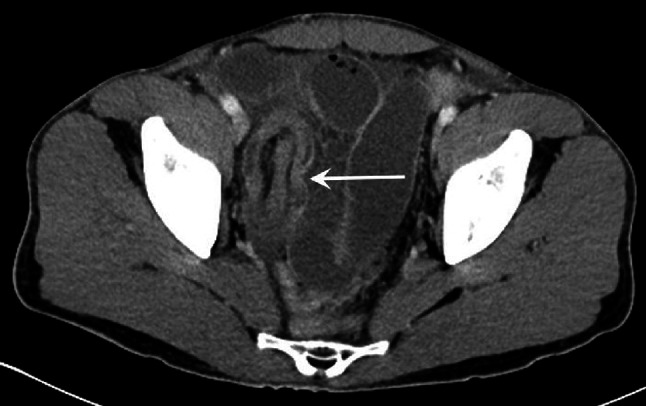
The intussusception (arrow) seen in the long segment of the ileum on axial CT images was treated surgically. It was determined to be consistent with Meckel’s diverticulum both surgically and histopathologically

**Fig. 4:**
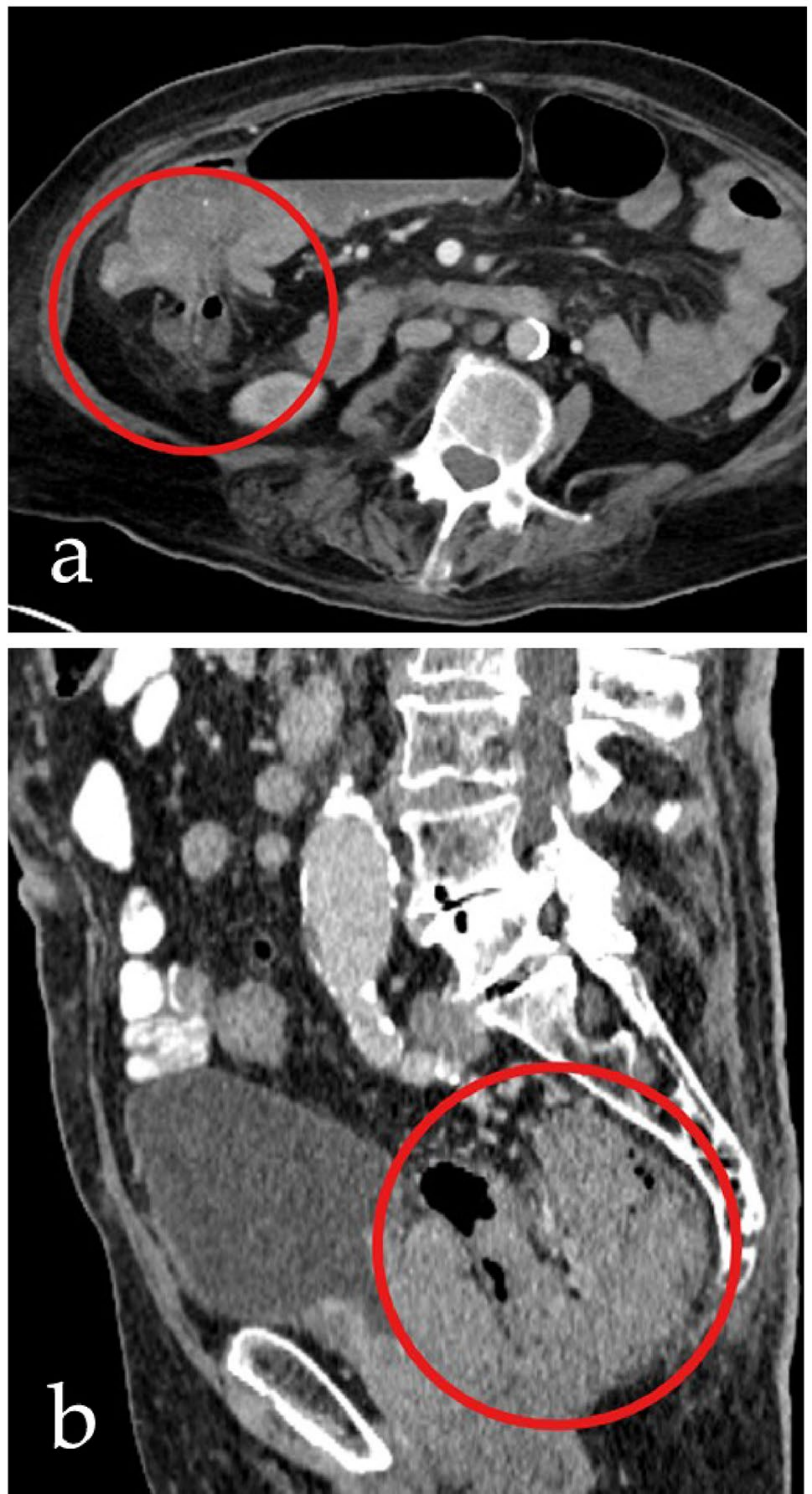
Contrast-enhanced CT images demonstrating adult intussusceptions secondary to malignant lead points. In panel (a), an axial image reveals a large, heterogeneous soft-tissue mass in the cecum (red circle) confirmed as an adenocarcinoma. In panel (b), a sagittal reconstruction shows a malignant mass in the rectum (red circle) acting as the lead point for the intussusception

**Fig. 5:**
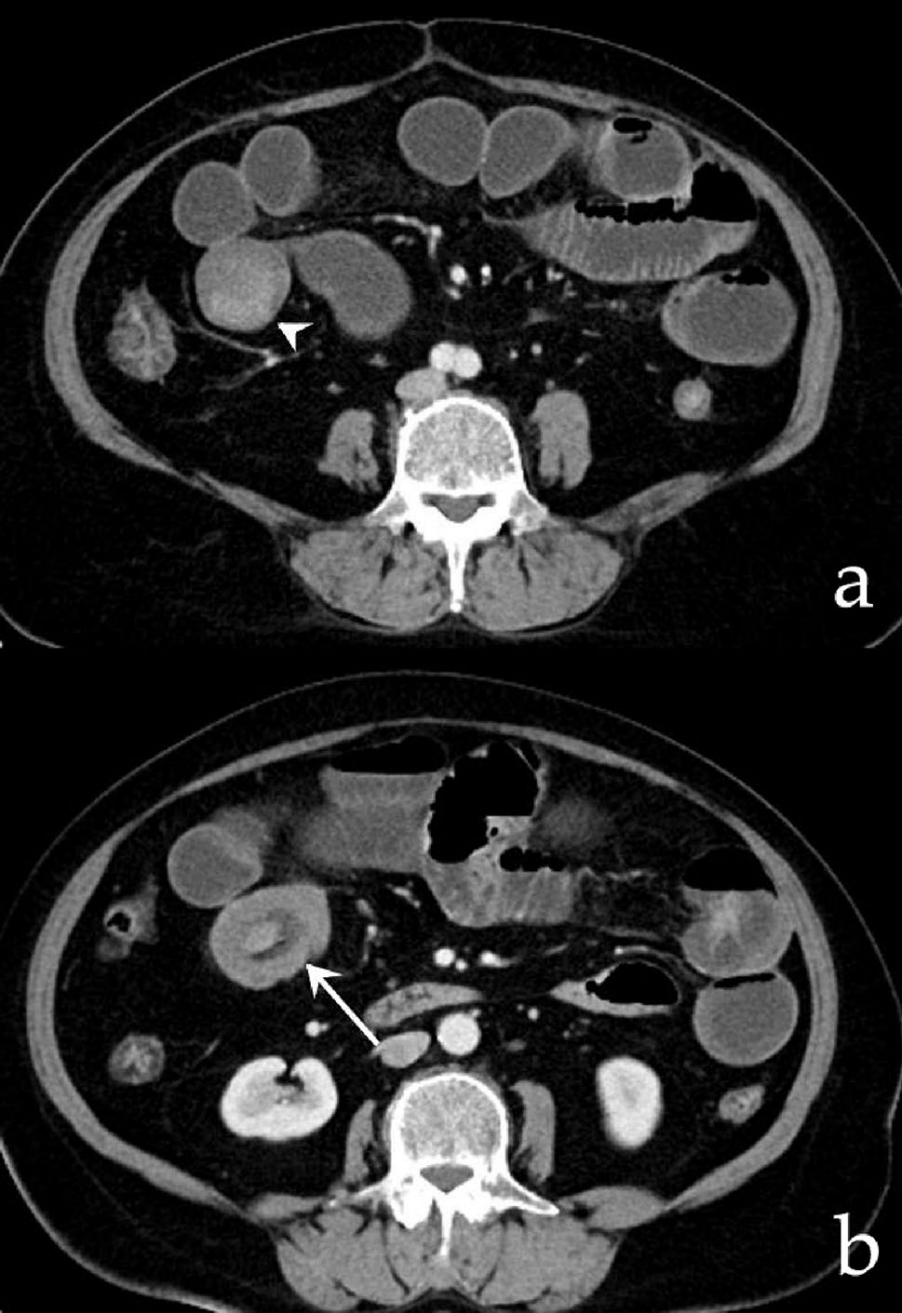
Axial CT images show wall thickening identified as the leading point in the ileal segment (a, arrowhead) and the resulting intussusception (b, arrow). Histopathologically, it was confirmed as melanoma metastasis

The majority of intussusceptions were located in the small bowel, with the jejunum being the most frequent site (*n* = 116, 77.3%), followed by the ileum (*n* = 17, 11.3%). Colonic or multisegmental involvements were rare (Table 1). A pathological lead point was identified in only 17 patients (11.3%), while no structural lesion was detected in the remaining 133 patients (88.7%). Five of the patients with lead points had benign lesions such as lipoma (Fig. 2) and Meckel’s diverticulum (Fig. 3). While the other 12 patients had malignancies such as adenocarcinoma (Fig. 4), malignant melanoma metastasis (Fig. 5), sarcoma infiltration). These patients were followed up with radiological and surgical consultation. Patients suspected of having malignancy underwent surgery, and their diagnoses were confirmed histopathologically. The mean length of the intussuscepted segment was 3.60 ± 1.92 cm (median: 3.00 cm; IQR: 2.25–4.50).

### Follow-up outcomes

**Fig. 6:**
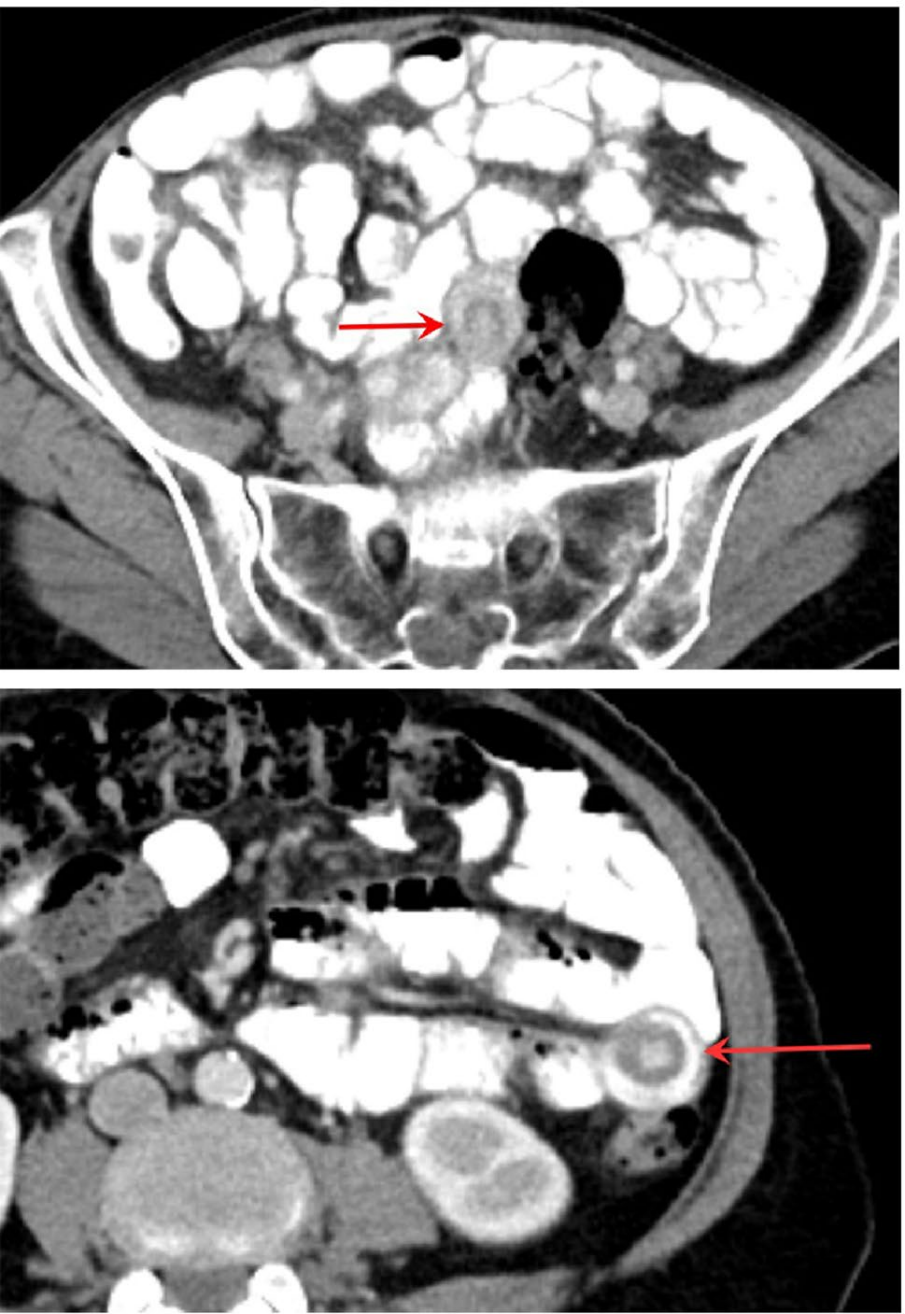
Axial contrast-enhanced CT images illustrating examples of transient enteroenteric intussusceptions. The red arrows highlight the characteristic “target sign” of the intussuscepted small bowel loops. In these cases, subsequent follow-up imaging confirmed the complete spontaneous resolution of the lesions without any surgical intervention, supporting their benign and transient physiological nature

Follow-up imaging was available for 59 patients to assess the evolution of the intussusception. The mean time interval between the initial and control imaging was 262.65 ± 403.22 days. On follow-up, the intussusception had spontaneously resolved in 44 patients (74.6%) (Fig. 6). Persistence of the finding was observed in 9 patients (15.3%), including 7 with the same lesion and 2 with a new intussusception in a different segment. Six patients (10.2%) underwent surgical intervention.

### Analysis of factors associated with persistence

We evaluated potential predictors for the persistence of intussusception in the subgroup of patients with follow-up imaging (*n* = 59). The analysis compared patients with persistent intussusception (*n* = 9) against those with resolution (*n* = 50) (Table 3).

**Table 3:** Comparison of clinical characteristics with persistence of intussusception on follow-up

		Persistence on Follow-up		*p* value
		Present *n* (%)	Absent *n* (%)	
Gender				0.471
	Male	5 (55.6)	34 (68.0)	
	Female	4 (44.4)	16 (32.0)	
Malignancy				0.236
	Yes	5 (55.6)	38 (76.0)	
	No	4 (44.4)	12 (24.0)	
Systemic Disease				0.999
	Yes	2 (22.2)	14 (28.0)	
	No	7 (77.8)	36 (72.0)	
History of Surgery				0.278
	Yes	7 (77.8)	27 (54.0)	
	No	2 (22.2)	23 (46.0)	
Ileus				0.284
	Present	1 (11.1)	1 (2.0)	
	Absent	8 (88.9)	49 (98.0)	
Lead Point (Lesion)				0.595
	Present	2 (22.2)	6 (12.0)	
	Absent	7 (77.8)	44 (88.0)	

“Present” group includes patients with persistent intussusception (n = 9). “Absent” group includes patients with resolution (n = 50)

There was no statistically significant difference between the two groups regarding age (*p* = 0.202) or the time interval to control imaging (*p* = 0.370). Although the median segment length was slightly higher in the persistent group (4.00 cm vs. 3.00 cm), this difference did not reach statistical significance (*p* = 0.184). When segment length was categorized (< 4 cm, 4–8 cm, > 8 cm), no significant association with persistence was found (*p* = 0.399) (Table 5).

**Table 4:** Comparison of quantitative variables with persistence of intussusception on follow-up

	Persistence on Follow-up		*p* value
	**Present** (*n* = 9)	**Absent** (*n* = 50)	
	*Median (Q1 - Q3)*	*Median (Q1 - Q3)*	
Age	44.00 (25.50–61.50)	50.50 (40.00–66.00)	0.202
Length of Intussuscepted Segment (cm)	4.00 (2.75–7.50)	3.00 (2.50–4.50)	0.184
Time to Follow-up Imaging (days)	196.00 (50.00–706.00)	109.50 (61.75–215.50)	0.370

**Table 5:** Comparison of segment length categories with presence of ileus and persistence

	Ileus		*p* value	Persistence on Follow-up		*p* value
	Present *n* (%)	Absent *n* (%)		Present *n* (%)	Absent *n* (%)	
Segment Length			0.390			0.399
< 4 cm	3 (37.5)	82 (58.2)		3 (33.3)	27 (54.0)	
4–8 cm	5 (62.5)	56 (39.7)		6 (66.7)	22 (44.0)	
> 8 cm	0 (0.0)	3 (2.1)		0 (0.0)	1 (2.0)	

Furthermore, clinical factors such as gender (*p* = 0.471), history of malignancy (*p* = 0.236), presence of systemic disease (*p* = 0.999), history of surgery (*p* = 0.278), presence of ileus at diagnosis (*p* = 0.284), or the detection of a lead point (*p* = 0.595) showed no statistically significant correlation with the persistence of the intussusception on follow-up imaging (Tables 4 and 5).

## Discussion

Our study evaluated the clinical and radiological characteristics of intussusceptions incidentally detected in abdominal CT examinations in adult patients. The data we obtained support the conclusion that the vast majority of adult intussusceptions are benign, transient, and self-limiting physiological processes. Contrary to classical information in the literature, it has been observed that parameters such as malignancy history or segment length are not statistically significant predictors of lesion persistence.

Adult intussusceptions have historically been considered a surgical pathology, and it has been reported that the likelihood of an underlying lesion (lead point) is 80–90%. [1, 12]. However, with the widespread adoption of multi-slice CT technology, these rates have been observed to change dramatically in recent series. In our study, the detectable pathological lead point rate was limited to 11.3%. This finding suggests that most invaginations encountered in modern radiology practice lack a pathological basis and that the older literature is based more on symptomatic surgical series, and therefore does not fully reflect the current asymptomatic population [8]. Indeed, even in recent literature, surgical case series continue to highlight underlying neoplasms and mechanical bowel obstruction as the primary features of adult intussusception [19].

One of the findings of our study is the high rate of spontaneous resolution of intussusceptions. Spontaneous resolution of intussusception was observed in 74.6% of cases undergoing follow-up imaging. This high resolution rate indicates that the majority of detected lesions are physiological phenomena (transient intussusception) associated with temporary peristaltic dysrhythmias rather than true obstructive pathology [20]. Indeed, the fact that 77.3% of our cases were localized in the jejunum segment, where peristaltic activity is most intense, and that only 4.7% of patients presented with ileus supports this physiological mechanism hypothesis [21]. In adult intussusception, the clinical spectrum appears to evolve into a chronic or incidental process.

One of the most controversial issues in clinical management is what approach should be taken in patients with a known history of malignancy. The general consensus is that intussusceptions detected in cancer patients may be metastatic and require aggressive management [22, 23]. However, in our study, although 40.0% of patients had a history of malignancy, no statistically significant relationship was found between the presence of malignancy and the persistence of intussusception. This data suggests that intussusception detected in patients with a history of malignancy may be incidental rather than metastatic, provided that no additional mass is seen on CT. Therefore, a history of malignancy alone may not justify aggressive surgical or invasive diagnostic procedures.

The length of the invaginated segment as a radiological marker is frequently discussed in the literature. Previous studies have argued that lesions above the 3.5 cm threshold are more likely to be pathological and require surgery [12, 15, 24]. However, our study found no statistically significant difference between segment length and the persistence of intussusception. In our series, where the average segment length was 3.6 cm, even long-segment lesions were observed to disappear during follow-up. This calls into question the reliability of segment length alone as a decision-maker. Similarly, the presence of systemic diseases and demographic factors such as gender or age were also found to be insufficient in predicting the prognosis of intussusception.

High spontaneous recovery rates suggest that current follow-up protocols need to be reviewed. The inability to identify a definitive biomarker or radiological marker to determine which patients require follow-up; suggests that radiological follow-up may not always be necessary in asymptomatic patients with short segments and no obvious mass on CT (especially considering the high negative predictive value of CT in ruling out lead point), and that clinical follow-up may be sufficient [2, 10]. The fact that the proportion of patients undergoing surgery in our series remained at a low level of 10.2% supports the idea that the phrase “surgical consultation is recommended” used in radiology reports could be replaced with “possible transient intussusception; clinical follow-up is recommended.”

Our study has several limitations that should be acknowledged. First, its retrospective and single-center design may introduce selection bias and limit the generalizability of the findings. Second, follow-up imaging was not available for all patients in the cohort, which might obscure the ultimate clinical outcome of those without control scans. Furthermore, the lack of histopathological correlation for patients managed conservatively prevents the definitive identification of the underlying etiology in resolved cases. Despite these limitations, our study has notable strengths. The inclusion of a relatively large cohort for a rare adult pathology, combined with detailed clinical-radiological correlations and the assessment of follow-up outcomes, provides valuable real-world evidence that challenges traditional surgical dogmas.

Future research should focus on prospective, multicenter studies with standardized clinical and radiological follow-up protocols to establish clear, evidence-based management guidelines. Additionally, the integration of advanced imaging analytics, radiomics, or artificial intelligence could be explored in future studies to better differentiate transient physiological intussusceptions from those harboring a malignant lead point at the initial CT examination.

## Conclusion

In conclusion, the vast majority of adult intussusceptions incidentally detected on CT are benign, short segment, jejunal predominant processes that demonstrate spontaneous resolution. Traditional predictive factors, such as a history of malignancy or the length of the intussuscepted segment, appear insufficient to foresee the persistence of the lesion. For asymptomatic patients lacking a distinct pathological lead point or clinical signs of obstruction on CT, a conservative approach prioritizing clinical observation should be considered to avoid unnecessary radiation exposure and surgical morbidity.

## Data Availability

No datasets were generated or analysed during the current study.
